# The Treatment of Cervical Erosion, with Special Reference to the "Pre-Cancerous" Cervix
1A Paper read at the Bath and Bristol Branch of the British Medical Association, May 26th, 1926.


**Published:** 1926

**Authors:** H. J. Drew Smythe

**Affiliations:** Assistant Obstetric Physician and Gynæcologist, Bristol General Hospital


### THE TREATMENT OF CERVICAL EROSION, WITH SPECIAL REFERENCE TO THE "PRE-CANCEROUS" CERVIX.[1]

BY

H. J. Drew Smythe, M.S., F.R.C.S. Eng.,
*Assistant Obstetric Physician and Gynæcologist,*
*Bristol General Hospital.*

---

Erosion of the cervix uteri is one of the commonest of gynæcological conditions seen, both in hospital and in general practice. Practically every other patient examined in a gynæcological out-patient department is found to be suffering from this condition in either a mild or a severe form. Sometimes it is but part of a general endocervicitis and endometritis, but in many cases it is the only abnormal condition present.

First I must refer briefly to the so-called "congenital erosion of the cervix," a term which is in a large number of cases a misnomer. In the true congenital erosion there is an overgrowth of the columnar epithelium at the expense of the squamous epithelium covering the cervix around the external os. This gives rise to an absolutely circular velvet patch surrounding the os, which is often seen in the new-born and may persist in adolescence. This patch becomes a ready focus for infection, and may account for the persistence of some cases of vulvo-vaginitis in children.

In a large number of cases, however, the so-called "congenital" erosion is due to a gonococcal infection of the cervix. In this condition the erosion is not

[1] A Paper read at the Bath and Bristol Branch of the British Medical Association, May 26th, 1926.

absolutely circular and often gives rise to small localised granulomata, inflammatory in origin.

Few cases of true congenital erosion require treatment, but if it is associated with leucorrhœa then one or two applications of pure carbolic acid may be made to the cervix. This may necessitate a preliminary incision of the hymen under local anæsthesia, but in the majority of cases the application may be made through a glass tube by means of a piece of wool on a wooden holder.

If the erosion is due to gonorrhœal infection (and this applies to any gonorrhœal cervical infection), the best treatment is local application of flavine or mercurochrome to it, after it has been cleansed of mucous discharge by means of a solution of sodium bicarbonate or weak liquor potassæ. The vagina and fornices are afterwards packed lightly with ribbon gauze soaked in flavine or mercurochrome.

We come now to the treatment of the common erosion of the cervix, which in practically all cases results from infection of a laceration occurring during labour. In these cases the treatment depends on three things, the age of the patient, the extent and kind of erosion, and the co-existence or not of other uterine disease.

In a simple case of erosion, for example that which is limited to the laceration of a recent confinement, and does not spread beyond this over the squamous-lined cervix, astringent douches will in the majority of cases effect a cure. Of these lead acetate, alum or zinc sulphate are the best, and these should not be given hot but nearly cold in order to get the best effect. At the same time attention should be paid to treatment of the general health of the patient, by regulation of the bowels, rest and suitable tonics, especially iron. If astringent douches fail to effect a cure, then repair of the cervical tear by operation is to be advised; the scar tissue and erosion are excised by a V-shaped incision and the edges of the V sutured together.

The application of strong caustics, especially repeated application, to the cervix, is not only inadvisable, but actually harmful. They cause deep sloughing with consequent formation of cicatricial tissue which leads to trouble in later pregnancies, and may be the starting-point in the formation of carcinoma of the cervix. In support of this statement may be quoted the case of Mrs. W., upon whom I operated in August, 1924.

This patient was an actress, 2-para, aged 35, who had suffered for several years from a vaginal discharge, and had for the six months previous to coming under my care been treated with applications of zinc chloride to the cervix. When I saw her she had an extensive squamous-celled cervical carcinoma, which on removal was seen to be growing from an area of dense cicatricial tissue. In my opinion this carcinoma was started by the application of strong caustic.

If the erosion has extended beyond the laceration, and especially if it is of the papillary type, thorough removal of the eroded area, either by curettage or excision with subsequent suture, is advisable. Even if the patient does subsequently become pregnant the cervix, after excision and suture, dilates quite evenly and does not tend to tear much more readily than the normal cervix.

Attention is particularly directed to a class of case in which the patient is around the menopausal age and complains of a continuous muco-purulent discharge. On examining the cervix it is found to be covered, or partially so, with an extensive proliferative erosion which bleeds slightly on examination. There is no definite ulceration and no macroscopic sign of carcinoma.

In other areas of the cervix partial healing of the erosion may have taken place, leaving a hard surface studded with ovulæ. I class all these cases as "pre-carcinomatous," that is, cases in which a carcinoma may arise and is indeed very likely to arise. In these circumstances I recommend a total hysterectomy, with conservation of one or both ovaries, depending on their condition. To many this may seem a too drastic and unnecessary course, so I will endeavour to justify it.

It is universally recognised that carcinoma of the cervix arises in the large majority of cases in women who have borne children. In most if not in all these patients there has been a preliminary cervical laceration. Commencing thus, the process continues to the formation of an erosion to which sepsis is added, the result being a chronically infected surface constantly bathed with pus and subject to perpetual irritation, conditions recognised as ideal for the development of carcinoma. Then again, in the healing process the columnar epithelium being replaced and invaded by the squamous epithelium, in the menopausal age 45–50 and after, there seems to be a loss of tissue balance; the squamous epithelium piles itself up and often breaks through the basement membrane, a carcinoma resulting.

It is impossible to discern with the naked eye the early carcinomatous changes, and even if one takes a section of the erosion one may not hit on the one small area of commencing carcinoma.

As an example of this, we have the case of Mrs. K., whom I first saw in November, 1925. She had at this time a typical pre-carcinomatous cervix. I advised pan-hysterectomy, to which she agreed; but she did not come in when sent for, and did not report again until six months later, when there was present a carcinoma visible to the naked eye.

As an alternative to pan-hysterectomy, there is amputation of the cervix; but by this means one does not remove all the cervical tissue, and a number of growths arise at the level of the internal os. Further, the body of the uterus remains, which in these cases is nearly always in a condition of chronic endometritis and metritis. Finally, at this age, *i.e.* around the menopause, the uterus is a functionless and useless organ.

The operative risk of total hysterectomy is extremely small, and even amputation of the cervix has a small operative risk. I have performed total hysterectomy for this type of case thirty-six times during the last twelve months, in hospital and private practice, without a single death. In my opinion the death rate from carcinoma of the cervix will be enormously lessened by radical operation on these pre-carcinomatous cases. It is better to remove a small number of functionless uteri, which may not develop carcinoma, than to allow a large number to go on to the formation of carcinoma, which can only be removed by an extensive operation with a 40 per cent. mortality.

